# Drug and Cell Type-Specific Regulation of Genes with Different Classes of Estrogen Receptor β-Selective Agonists

**DOI:** 10.1371/journal.pone.0006271

**Published:** 2009-07-17

**Authors:** Sreenivasan Paruthiyil, Aleksandra Cvoro, Xiaoyue Zhao, Zhijin Wu, Yunxia Sui, Richard E. Staub, Scott Baggett, Candice B. Herber, Chandi Griffin, Mary Tagliaferri, Heather A. Harris, Isaac Cohen, Leonard F. Bjeldanes, Terence P. Speed, Fred Schaufele, Dale C. Leitman

**Affiliations:** 1 Departments of Obstetrics, Gynecology and Reproductive Sciences, Cellular and Molecular Pharmacology, Center for Reproductive Sciences, University of California San Francisco, San Francisco, California, United States of America; 2 Bionovo Inc., Emeryville, California, United States of America; 3 Center for Statistical Sciences & Department of Community Health, Brown University, Providence, Rhode Island, United States of America; 4 Women's Health and Musculoskeletal Biology, Wyeth Research, Collegeville, Pennsylvania, United States of America; 5 Department of Nutritional Science and Toxicology, University of California, Berkeley, California, United States of America; 6 Department of Statistics, University of California, Berkeley, California, United States of America; and Division of Bioinformatics, The Walter and Eliza Hall Institute of Medical Research, Parkville, Victoria, Australia; 7 Department of Medicine, University of California San Francisco, San Francisco, California, United States of America; Ecole Normale Supérieure de Lyon, France

## Abstract

Estrogens produce biological effects by interacting with two estrogen receptors, ERα and ERβ. Drugs that selectively target ERα or ERβ might be safer for conditions that have been traditionally treated with non-selective estrogens. Several synthetic and natural ERβ-selective compounds have been identified. One class of ERβ-selective agonists is represented by ERB-041 (WAY-202041) which binds to ERβ much greater than ERα. A second class of ERβ-selective agonists derived from plants include MF101, nyasol and liquiritigenin that bind similarly to both ERs, but only activate transcription with ERβ. Diarylpropionitrile represents a third class of ERβ-selective compounds because its selectivity is due to a combination of greater binding to ERβ and transcriptional activity. However, it is unclear if these three classes of ERβ-selective compounds produce similar biological activities. The goals of these studies were to determine the relative ERβ selectivity and pattern of gene expression of these three classes of ERβ-selective compounds compared to estradiol (E_2_), which is a non-selective ER agonist. U2OS cells stably transfected with ERα or ERβ were treated with E_2_ or the ERβ-selective compounds for 6 h. Microarray data demonstrated that ERB-041, MF101 and liquiritigenin were the most ERβ-selective agonists compared to estradiol, followed by nyasol and then diarylpropionitrile. FRET analysis showed that all compounds induced a similar conformation of ERβ, which is consistent with the finding that most genes regulated by the ERβ-selective compounds were similar to each other and E_2_. However, there were some classes of genes differentially regulated by the ERβ agonists and E_2_. Two ERβ-selective compounds, MF101 and liquiritigenin had cell type-specific effects as they regulated different genes in HeLa, Caco-2 and Ishikawa cell lines expressing ERβ. Our gene profiling studies demonstrate that while most of the genes were commonly regulated by ERβ-selective agonists and E_2_, there were some genes regulated that were distinct from each other and E_2_, suggesting that different ERβ-selective agonists might produce distinct biological and clinical effects.

## Introduction

Estrogens exert their biological effects by interacting with two known ERs, ERα and ERβ [1], [2], [3], [4]. ERs are involved in development of the reproductive tract and regulation of reproductive processes [5]. In addition to their role in reproduction, ERs also have important roles in the breast, bone, brain and the cardiovascular system [1], [2], [3], [4]. Studies with ERα and ERβ knockout mice demonstrated that ERα is required for the development of certain tissues in the reproductive tract and mammary gland [6]. ERβ knockout mice (βERKO) show other defects. There are fewer corpora lutea in the βERKO mice, which likely accounts for the observation that these mice are subfertile [7]. In luminal mammary epithelial cells of βERKO mice there was a widespread increase in the proliferation marker, Ki-67, suggesting that ERβ is important for terminal differentiation of mammary epithelial cells [8]. Prostate and myelogenous hyperplasia have been observed in βERKO mice [9], [10]. These mice also show a loss of anxiety [11] and spatial learning [12], and developed depression-like behavior [13]. These observations support a role for ERβ in behavior, mood and affective disorders.

Estrogens have been used extensively to treat menopausal symptoms and osteoporosis in postmenopausal women. The Women's Health Initiative (WHI) trial found that the risks outweighed the benefits of hormone therapy (HT) [14], [15], [16], [17], [18]. It is now thought that some adverse effects of HT observed in the WHI were due to an older subject population in the trial [19], [20]. However, there remains an intense effort to discover safer estrogens that selectively regulate ERα or ERβ, as alternatives to the estrogens currently used in HT regimens that non-selectively regulate both ERs.

ERβ-selective estrogens might be more desirable for HT than ERα-selective estrogens, because studies indicate that ERα mediates cell proliferation that contributes to breast and endometrial cancer whereas ERβ generally is thought to counteract ERα-dependent cell proliferation and tumor formation [21], [22], [23]. The first reported ERβ-selective estrogen synthesized and studied was diarylpropionitrile (DPN). DPN has a 70-fold higher *in vitro* binding affinity and 170-fold higher potency in transcription assays with ERβ compared to ERα [24]. Other ERβ-selective ligands have been synthesized in both academic and industrial settings, of which ERB-041 is among the most studied [7], [25]. In addition to synthetic ERβ ligands, a plant extract, MF101 [26] and a flavanone derived from a single plant in MF101, liquiritigenin [27] are highly ERβ-selective compounds.

Studies with ERβ-selective compounds indicate that there are at least three classes of ERβ-selective agonists. ERB-041 is the prototype of a ligand that is an ERβ-selective binder, because it binds to ERβ with a much higher affinity than ERα. In contrast, we showed that MF101 and liquiritigenin bind similarly to ERα and ERβ, but do not regulate gene transcription in the presence of ERα or stimulate uterine growth or breast cancer tumor formation in mouse models [27]. These studies established that some ligands can act as highly ERβ-selective transcriptional activators, even though they bind non-selectively to both ERα and ERβ. A third class of ERβ-selective agonists is represented by DPN, which is selective by a combination of preferential binding to ERβ and increased transcriptional activity [24]. An unanswered question is whether different ERβ-selective agonists produce biological effects that are distinct from each other and non-selective ER agonists used in HT, such as estradiol. To investigate this issue, we determined if these ERβ-selective compounds regulate the same or different genes.

## Materials and Methods

### Reagents

MF101, liquiritigenin and nyasol were obtained from Bionovo (Emeryville, CA). ERB-041 was obtained from Wyeth (Collegeville, PA). DPN was obtained from Tocris (Ellisville, MO). Estradiol was obtained from Sigma-Aldrich Chemical Co. (St. Louis, MO). All other compounds were obtained as previously described [27], [28], [29].

### Cell lines and culture

Tetracycline-inducible U2OS-ERα and U2OS-ERβ cells were characterized and maintained as previously described [30]. U2OS, Caco-2, HeLa, and Ishikawa cells were obtained from the UCSF cell culture facility and maintained as previously described [28], [31]. All experiments were done with cells containing 5% charcoal-stripped fetal bovine serum.

### Förster resonance energy transfer (FRET)

**Figure 1:**
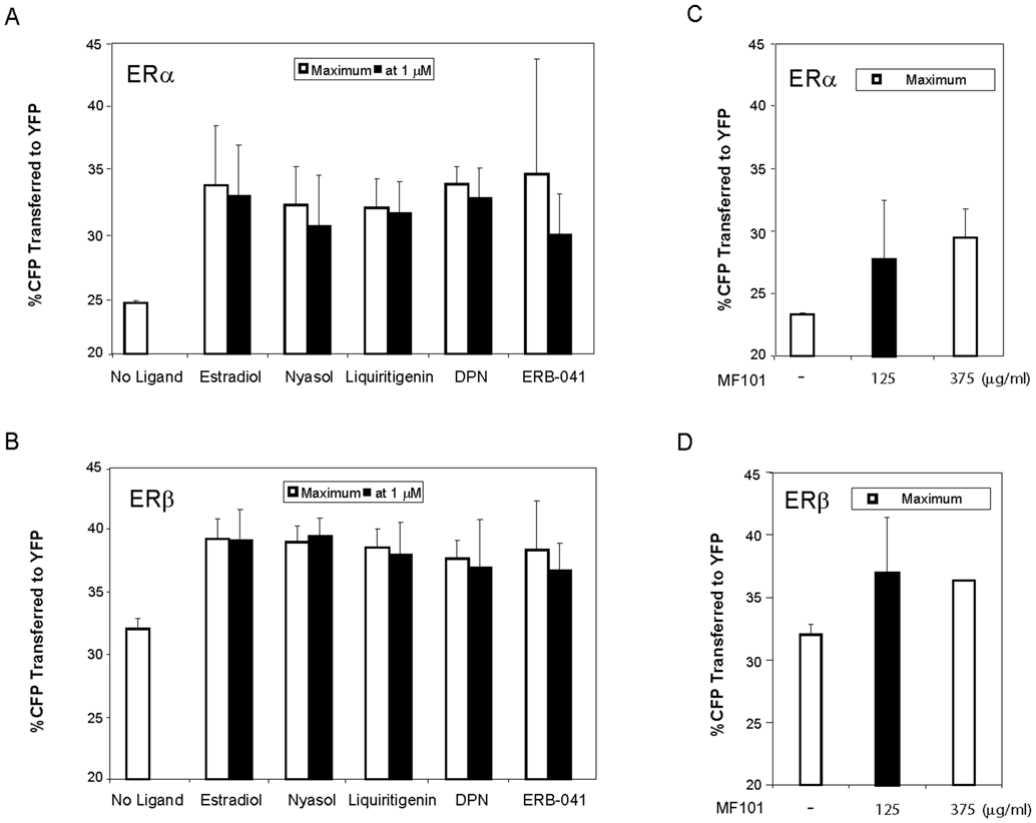
ERβ-selective compounds produce conformational changes with both ERα and ERβ at saturating levels. U2OS cells were transfected with CFP-ERα-YFP (A, C) or CFP-ERβ-YFP (B, D) and then treated with 1 µM estradiol, nyasol, liquiritigenin, DPN, ERB-041 (A, B, solid bars) or with 125 µg/ml MF101 (C, D solid bars). Cells were imaged from 30 to 40 minutes following ligand addition. Percentage of CFP energy transferred to YFP was not significantly different comparing to its maximum values obtained in dose-response experiments (open bars). The amounts of energy transferred at the concentration used in the gene profiling studies (1 µM, closed bars) were not statistically different than the amounts at saturation, with the exception that 1 µM ERB-041, which did not saturate ERα (A). Error bars represent the SD of four independent experiments in which FRET was collected at 1 µM; the dose response curves were collected in two independent experiments.

U2OS cells (*n* = 500,000) were plated into six-well dish containing a borosilicate glass coverslip and grown in phenol red-free DMEM/F12 media supplemented with 5% charcoal-stripped fetal bovine serum and 2 mM glutamine. The following day the cells were transfected with 500 ng/well of CFP-ERα-YFP [32] or CFP-ERβ-YFP [26] using Lipofectamine™ 2000 according to manufacturer's protocol (Invitrogen, Carlsbad, CA). After 6 h the medium was replaced with complete medium containing 10% stripped fetal bovine serum, 2 mM glutamine, 50 U/ml penicillin, 50 µg/ml streptomycin and the cells were incubated overnight. One day after transfection cells were treated with the indicated amounts of ligand for 30 minutes before image collection. Within each independent experiment, an average of 124 cells were collected for each ligand at each concentration and the amount of FRET averaged by comparing the amounts of fluorescence in the acceptor bleedthrough corrected FRET channel to the amount in the Donor channel; the conversion of these values to the percentage of Energy transferred from CFP to YFP was done using the calibration methods we have previously described [33]. For each ligand, the dose response curves were conducted twice on independent days and presented at the mean+/−range (Figure 1, open bars). Measurements at 1 µM of ligand were repeated on four independent days and presented as the mean+/−standard deviation (Figure 1, closed bars). In total, FRET measurements were collected from 35,396 cells expressing CFP-ERα-YFP or CFP-ERβ-YFP and from an additional 4,432 control cells expressing ERα or ERβ attached to CFP or YFP alone.

### Microarrays

U2OS-ERα and U2OS-ERβ cells were maintained in 5% charcoal-stripped fetal bovine serum and plated in 6-well plates. When the cells reached 80% confluent, they were treated with 1 µg/ml doxycycline for 12 h to induce ERs. The cells were then treated with 10 nM E_2_, 125 µg/ml MF101, or 1 µM liquiritigenin, nyasol or DPN for 6 h at 37 C. Total cellular RNA was isolated with the Aurum RNA isolation kit (Bio-Rad, Hercules, CA) per the manufacturer's protocol. RNA was first quantified by standard spectrophotometry, and then qualitatively evaluated by capillary electrophoresis employing the Bio-Rad Experion system (Hercules, CA). Biotin-labeled cRNA samples were prepared with 750 ng of total RNA template. Following synthesis and purification, the biotin-labeled samples were evaluated by both 260/280 absorbance spectrophotometry and capillary electrophoresis. The final labeled cRNA samples were hybridized overnight against Human genome HG U133A-2.0 GeneChip arrays containing more than 22,200 probe sets (Affymetrix, Santa Clara, CA) or 48,000 transcripts HumanWG-6 BeadChip (Illumina, San Diego, CA) arrays. For the U133A-2.0 GeneChips the array hybridizations, washing, staining, as well as scanning were performed by the J.D. Gladstone Genomics Core, (San Francisco, CA), whereas the Ilumina microarrays were processed at the UCSF Genomics Core. The drug studies were done with the U133A-2.0 GeneChips and the four cell type study was done with WG-6 BeadChips. Same batch of microarrays were used for all treatments and most treatments were done in triplicate except for NYA treatment in U2OS-ERα samples in 2 replicates, and E2, MF101, and LIQ treatment in U2OS-ERβ samples in four replicates.

### Microarray data analysis

The Affymetrix expression arrays were pre-processed using a variant of GCRMA [34]. The microarrays were preprocessed with a procedure similar to GCRMA, except that the background adjustment step is modified. Instead of using the probe sequence to predict non-specific binding (as in GCRMA), the non-specific binding for each probe is estimated from a database composed of hybridization data on the same platform of microarrays used in a variety of experiments. The new procedure is therefore dubbed dbRMA. Background parameters were estimated for each probe separately in dbRMA and avoided borrowing information across probes sharing similar but not identical sequences. More specifically, the probe intensity across all the samples in the database was modeled as a mixture distribution with the first component as background and estimated using normal approximation. Assessment on calibration data (Affymetrix Latin Square experiment) showed better accuracy of background parameters compared to those predicted by sequence. The normalization and summarization steps in the preprocessing procedures remain the same as GCRMA. The details of dbRMA procedure will be presented in a separate manuscript.

The Illumina expression arrays were pre-processed using lumi package [35]. The differential expression analysis was performed using limma package [36]. These packages are all available in R/BioConductor. For drug screen data, probesets were selected for further analysis if the fold change was greater than 2 and multiple testing adjusted p-value using Benjamini and Hochberg procedure (BH-adjusted p-value) was less than 0.05 [37]. For the three cell line data, fold change threshold 1.5 was used. The heatmaps of log intensities of genes across different experiments were produced using Cluster and TreeView software [38]. Cluster software was used to perform the hierarchical clustering based on Pearson correlation coefficients (PCC) to find clusters of genes with similar expression patterns. TreeView was then used to visualize the clusters and produce the figures.

### Functional enrichment analysis of target genes

To elucidate the biological processes of target genes, we searched enriched GO annotations using GOstat software [39]. For each annotated GO term, GOstat counted the number of overlapping genes from the input gene list, and compared it with the one expected from a reference list (GO annotation human (http://www.ebi.ac.uk/GOA/human_release.html). Fisher's exact test was performed to compute a p-value for each GO category and BH-adjusted p-values were calculated. Results for significant GO “biological process” categories were reported. To compare the enriched GO terms cross different experiments, the scores 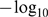 of BH-adjusted p-values for each GO term were combined into one table with GO terms shown in rows and different experiments shown in columns. Cluster and TreeView software [38] were then used to produce the GO charts.

### Western blot analysis

Caco-2, HeLa and Ishikawa cells were infected with an adenovirus (100 MOI) that expresses LacZ or ERβ [21]. Total proteins (20 µg) from cells were separated with 4%–12% gradient Bis-Tris gels (Invitrogen). Proteins were transferred to polyvinylidene difluoride (PVDF) membranes (Perkin Elmer) and probed with anti-ERα (DAKO), or three monoclonal ERβ antibodies (GeneTex) followed by anti-mouse IgG conjugated with horseradish peroxidase (PharMingen) as previously described [30]. An ECL detection system (GE HealthCare) was used for protein detection.

### RNA extraction and quantitative real-time PCR

Caco-2, HeLa and Ishikawa cells were infected with an adenovirus (100 MOI) that expresses ERβ [21]. After 20 h, the cells were treated for 6 h with MF101 or LIQ. Total RNA was extracted with Aurum total RNA mini kit and cDNA synthesis was performed with the iScript cDNA synthesis kit (Bio-Rad, Hercules, CA). Real-time PCR analysis was performed in duplicates using iQ SYBR Green Mix with an iCycler thermal cycler (Bio-Rad, Hercules, CA). U2OS-ERα and U2OS-ERβ were treated with 1 µg/ml doxycycline for 12 h to induce ERs. The cells were then treated for increasing times with the drugs and real-time PCR was done using primers for keratin 19 (K19), A kinase (PRKA) anchor protein 1 (AKAP1), interleukin 17 receptor B (IL17RB). The sequences of primers used are listed in Table S1.

## Results

### ERβ-selective compounds produce conformational changes in both ERα and ERβ

One goal of this study was to compare the relative ERβ-selectivity of three classes of ERβ agonists and to determine if they produce similar effects on gene expression to each other and E_2_. The structures of the compounds are shown in Figure S1. ERB-041 is an ERβ-selective binder because it binds 200-fold greater to ERβ than ERα [40]. MF101, liquiritigenin and nyasol are ERβ-selective activators, because they bind similarly to ERα and ERβ, but activate genes only with ERβ [26], [27]. DPN is a combined ERβ-selective binder and activator because of greater binding to ERβ and transcriptional activity with ERβ [24]. For comparison, we chose to study the effects of these drugs on gene expression at saturating concentrations of the compounds. FRET was used to determine the concentration required for saturation of the ligands to ERα and ERβ. The amount of FRET between CFP and YFP attached on opposite termini of each ER was shown to be a measure of ligand binding in intact cells [26], [32], [41]. U2OS cells were transfected with CFP-ERα-YFP or CFP-ERβ-YFP [26], [32] and then treated with the compounds. All of the compounds produced a dose-dependent enhancement of FRET with both ERα and ERβ when added to the cell culture medium at concentrations ranging from 0.3 nM to 3 µM (data not shown). The maximal amount of energy transfer at saturating amounts of ligand is shown for ERα (Figure 1A, open bars) or ERβ (Figure 1B, open bars) and is compared to the amounts of energy transfer detected at the 1 µM concentration (closed bars). All compounds produced equivalent amounts of energy transfer, above the no ligand controls, with both ERα and ERβ when provided at saturating levels. Note that the large error bars for ERB-041 at ERα (Figure 1A, open bars) reflects the variations in the extrapolation of the dose-response because maximal energy transfer was not achieved at 3 µM ERB-041 (the highest concentration used). Thus at 1 µM, all compounds except ERB-041 were able to saturate both ERα and ERβ. Similarly 125 µg/µl of the crude MF101 extract was sufficient to activate both ERα (Figure 1C) and ERβ (Figure 1D). We previously showed that 1 µM liquiritigenin (LIQ) and 125 µg/µl MF101 was the concentration that maximally activated reporter genes [26], [27]. Furthermore, 1 µM of nyasol (NYA), ERB-041 and DPN produced a maximal activation of ERE-tkLuc with ERβ in transfection assays (Figure S2). Based on the transfection and FRET studies, 1 µM of each compound and 125 µg/µl of MF101 extract was used for the subsequent studies to establish the ER subtype-selectivity of each compound.

### MF101, liquiritigenin and ERB-041 are the most ERβ-selective compounds

**Figure 2:**
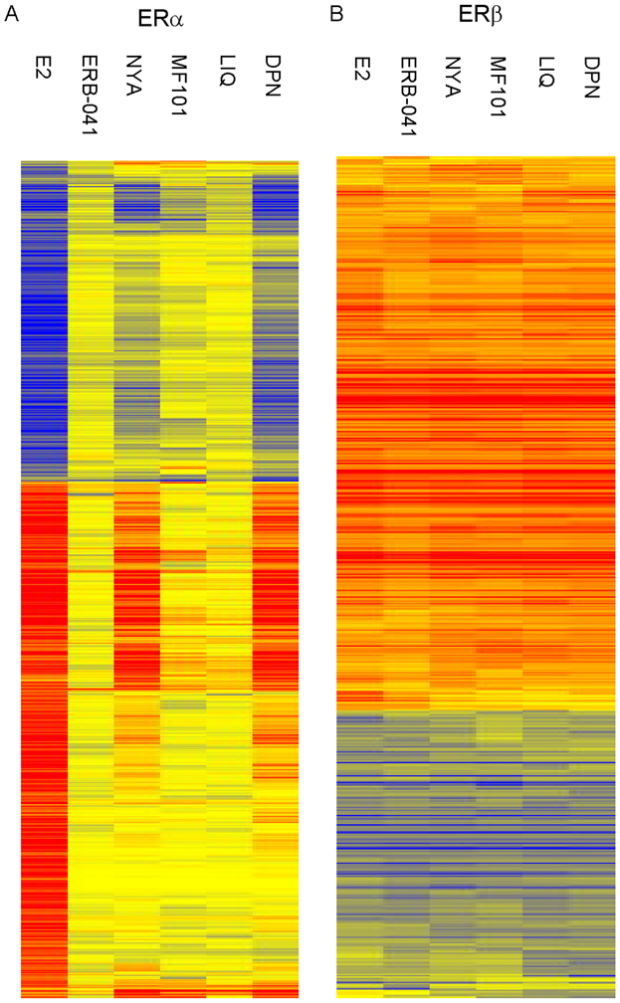
Heatmaps of genes regulated by different compounds in U2OS-ERα or U2OS-ERβ cells. Genes regulated by at least one of the compounds in U2OS-ERα (A) or U2OS-ERβ (B) cells are shown in rows. U2OS-ERα or U2OS-ERβ cells were treated for 6 h with vehicle, 10 nM E_2_, 125 µg/ml MF101, ERB-041, nyasol (NYA), liquiritigenin (LIQ) or (diarylpropionitrile) DPN. For each gene (row), entries with no fold change compared to vehicle control cells are colored yellow, relatively higher expression are colored with reds of increasing intensity, and relatively lower expression are colored with blues of increasing intensity.

To investigate the ERβ-selectivity of synthetic and natural compounds, we used the previously characterized human U2OS cells that are stably transfected with a doxycycline-inducible expression vector for ERα or ERβ [30]. After the cells were treated with doxycycline to induce ERs, they were treated with E_2_ and the plant-derived ERβ-agonists, MF101, NYA and LIQ, and the synthetic ERβ-agonists, DPN [24] and ERB-041 [40]. We previously showed that MF101 is a selective ERβ agonist despite being a complex, crude plant extract [26]. LIQ was isolated from *Glycyrrhizae uralensis* Fisch and is ERβ-selective [27]. NYA is a diphenylpentane norlignan that was purified from the plant *Anemarrhena asphodeloides* in MF101 and has ERβ-selectivity using transfection assays (data not shown). For each compound we defined a regulated gene to be activated by 2.0-fold or greater or repressed by 50% or greater and statistically different from the untreated control cells (BH-adjusted p-value<0.05). The regulated genes and magnitude of regulation in U2OS-ERα and U2OS-ERβ cells by each drug are found in Table S2. The heatmaps show the genes that are significantly regulated by the drugs compared to the control cells. The compounds produced a distinct pattern of regulated genes in the U2OS-ERα (Figure 2A) cells compared to U2OS-ERβ cells (Figure 2B). The non-ER selective agonist E_2_, which was used as a positive control, regulated 489 specific genes in the U2OS-ERα cells relative to the control cells (Table 1). In the U2OS-ERα cells, there were a total of 238 genes regulated by DPN and 152 genes regulated by nyasol. The Gene Ontology (GO) analysis showed that the major classes of genes commonly regulated in U2OS-ERα cells by E_2_, nyasol and DPN were involved in anatomical structure development, multicellular organismal process and developmental process (Figure S3). ERB-041 regulated 2 genes in the ERα cells, whereas LIQ and MF101 weakly regulated (between 2–3 fold) 3 and 16 genes in the ERα cells, respectively. These results demonstrate that relative to E_2_, only DPN and NYA showed ERα activity. In contrast, all the drugs regulated about 400 genes in the U2OS-ERβ cells (Table 1). The heatmap shows that overall the genes regulated by the ERβ agonists were similar to each other and to E_2_ (Figure 2B). By comparing the results in the U2OS-ERα and U2OS-ERβ cells the most ERβ-selective agonists at saturating levels were ERB-041, LIQ and MF101 followed by NYA, and then DPN.

**Table 1:** Summary of genes regulated for each compound in U2OS-ERα or U2OS-ERβ cells.

	ERα	ERβ	ERα and ERβ
ERB-041	2	379	0
LIQ	3	430	0
MF101	13	382	3
NYA	98	375	54
DPN	143	337	95
E2	489	200	236

Total genes regulated by the compounds, specifically in U2OS-ERα or U2OS-ERβ cells or in both cell types. Numbers are the probe set counts. The cells were treated for 6 h with 10 nM E_2_, 125 µg/ml MF101 or 1 µM of the other compounds. Microarrays were performed with U133A-2.0 GeneChips. Genes with fold change more than 2 and with BH-adjusted p-value< = 0.05 were considered.

**Figure 3:**
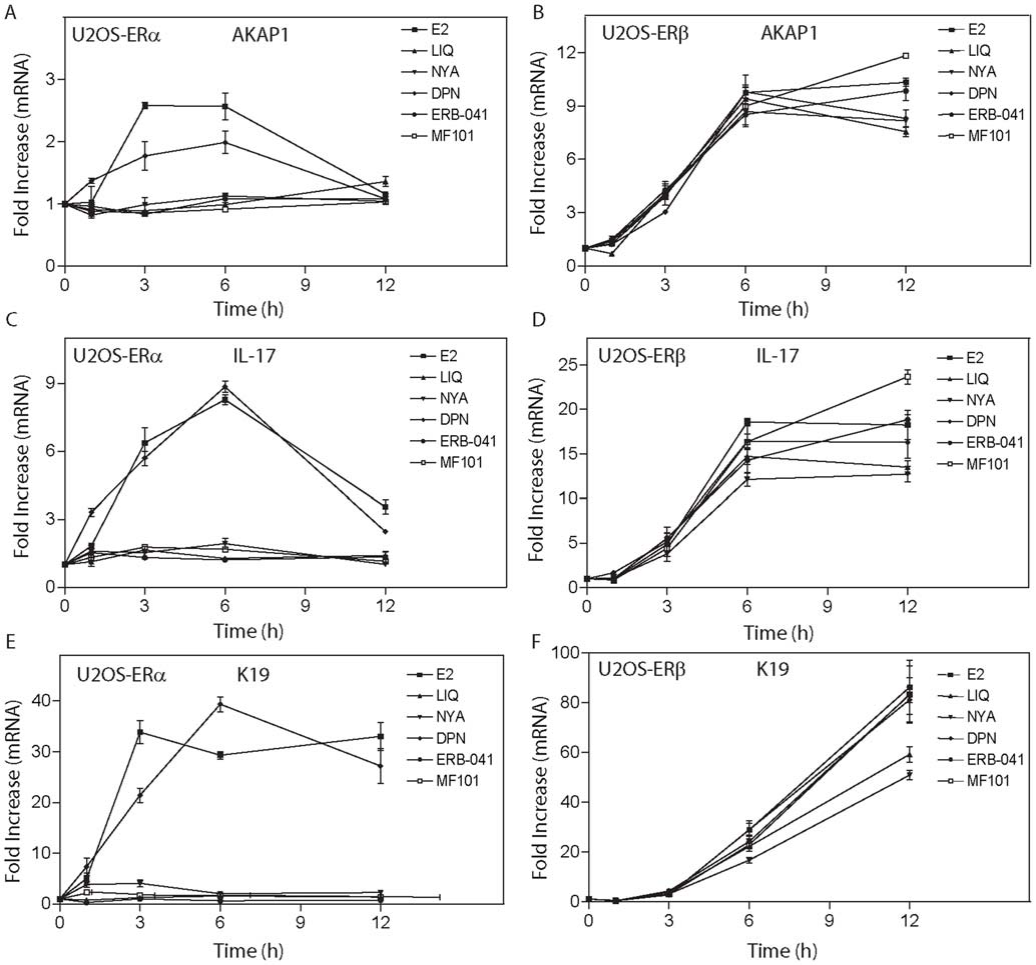
Time-course of gene regulation by E_2_ and ERβ-agonists in U2OS cells. U2OS-ERα or U2OS-ERβ cells grown in stripped fetal bovine serum were untreated or treated with doxycycline for 18 hours to induce ERs. The cells were then treated with E_2_ (10 nM), LIQ (1 µM), NYA(1 µM), DPN (1 µM), ERB-041(1 µM), and MF101 (125 µg/ml). Following treatment, mRNA levels for AKAP1, IL-17, and K19 were measured at 1, 3, 6 and 12 hours by real-time PCR in U2OS-ERα (A, C, and E, respectively) and U2OS ERβ (B, D, and F, respectively) cells. Each data point is the average of triplicate determinations. Error bars represent the mean±S.E.M.

To investigate that the possibility that the different genes regulated by ERα and ERβ were related to the 6 hour treatment time, we performed time-courses on three regulated genes (Figure 3). In the U2OS-ERα cells, E_2_ and DPN maximally activated AKAP1 (Figure 3A), IL-17 (Figure 3C), and K19 (Figure 3E) at 6 hour. No regulation was observed with other drugs at all time points. In contrast, all the drugs activated AKAP1 (Figure 3B), IL-17 (Figure 3D), and K19 (Figure 3F) in the U2OS-ERβ cells. The maximal activation of AKAP1 and IL-17 occurred at 6 hours, whereas K19 was maximally activated by the drugs at 12 h. All of drugs produced the maximal activation of these three genes at the same time-point in both U2OS-ERα and U2OS-ERβ cells. These findings indicate that the differences in regulation by drugs in the microarrays were not due to the selection of the 6 hour time-point.

### ERβ-selective compounds regulate some different genes in U2OS-ERβ cells

**Figure 4:**
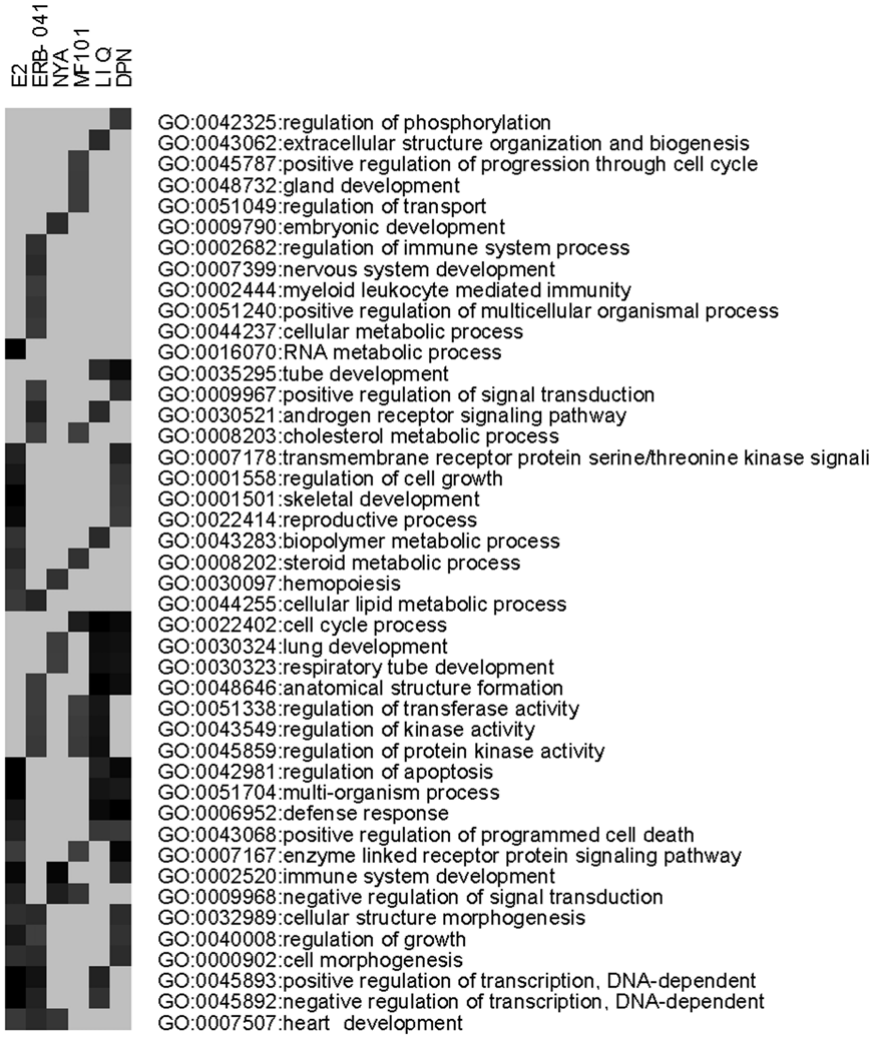
Analysis of biological processes differentially enriched among ERβ regulated genes between E2 and other compounds. Gene ontology (GO) terms showing significantly enriched in genes regulated by E_2_ or other compounds in U2OS-ERβ cells. A threshold 0.001 was used for selecting GO terms using BH-adjusted p-values. 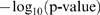 was used as an enrichment score. Darker shading denotes more significantly enriched GO terms, whereas the lightest gray implies the corresponding GO term is not significantly enriched. Only the GO terms significantly enriched in at least three conditions are shown.

**Figure 5:**
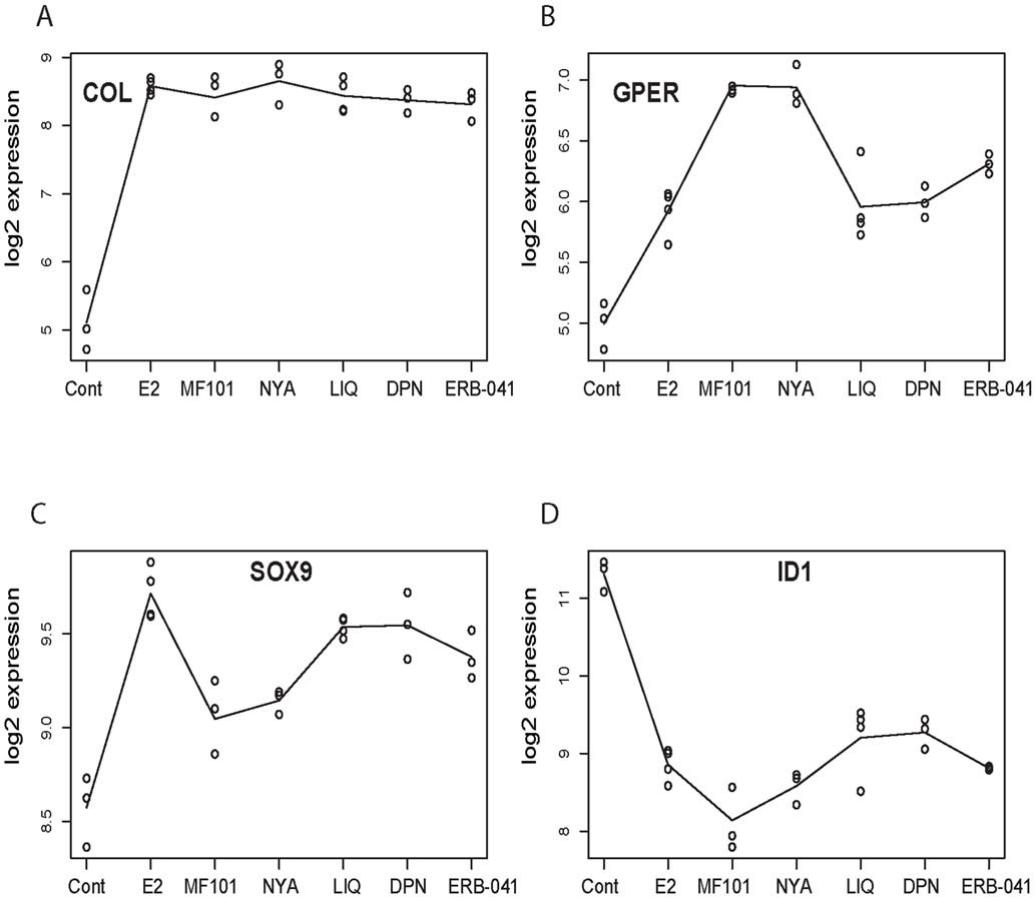
Gene expression profiles in ERβ cells for four regulated genes. In each plot, the y-axis is the log2 of expression intensity and the x-axis shows the drug index for the control, E_2_, MF101, NYA, LIQ, DPN, and ERB-041. The collagen, type XVIII, alpha 1 (COL) gene shows a large change compared to control and no expression change across different drugs with BH-adjusted p-value equal to 1. The other three genes show different pattern of gene expression profiles across the drugs with BH-adjusted p-values equal to 4e-09 for G protein-coupled estrogen receptor 1 (GPER), 3e-06 for inhibitor of DNA binding 1, dominant negative helix-loop-helix protein (ID1), and 1e-05 for SRY (sex determining region Y)-box 9 (SOX9). The p-values here are derived from the F-test comparing the log-intensities across different drugs.

Further analysis of the microarray data was done to determine if the three classes of ERβ-selective agonists regulate different genes in the U2OS-ERβ cells. Overall most of genes were commonly regulated with the ERβ-selective compounds (Table 2). The list of the regulated genes by each compound is found in Table S2. However, some genes were uniquely regulated by the ERβ-selective compounds (Table 2). The ERβ-selective agonists regulated more genes in common with each other compared to E_2_ in the U2OS-ERβ cells. The greatest difference in commonly regulated genes occurred with MF101 and E_2_, whereas LIQ and DPN showed no difference in the gene expression profiles. Some genes regulated by E_2_ in the ERβ cells were also different from those regulated by the ERβ-selective compounds. We performed GO analysis to determine what classes of genes were regulated similarly and differently by the ERβ agonists. Most of the classes of genes were regulated similarly, such as developmental process, multicellular organismal development, system development, organ development, biological regulation, and negative regulation of cellular process (Figure S4). However, some classes of genes were differentially regulated by the ERβ-selective drugs and E_2_ (Figure 4). For example, E_2_ uniquely regulated RNA metabolic process genes, whereas NYA regulated embryonic development genes, MF101 regulated gland development genes, LIQ regulated extracellular structure organization genes and biogenesis, and DPN regulated the regulation of phosphorylation genes (Figure 4). The magnitude of regulation by the drugs of several differentially regulated genes is shown in Figure 5. For comparison, the COL gene was regulated similarly by all the drugs (Figure 5A). The highest activation of the GPER gene was observed with MF101 and NYA (Figure 5B), and E_2_, LIQ, DPN and ERB-041 for the SOX9 gene (Figure 5C). The ID1 was repressed the most with E_2_, MF101, NYA and ERB-041 (Figure 5D). These results demonstrate that while most of the genes are commonly regulated there are some differences in class of genes regulated and the magnitude of regulation by the different drugs, which might be important in producing biological effects.

**Table 2:** Comparison of differentially expressed genes between compound pairs in U2OS-ERβ cells.

	E2	ERB-041	NYA	MF101	LIQ	DPN
E2	0	32	42	168	90	31
ERB-041	32	0	29	52	39	18
NYA	42	29	0	20	13	4
MF101	168	52	20	0	33	32
LIQ	90	39	13	33	0	0
DPN	31	18	4	32	0	0

Numbers are the probe set counts. Genes with fold change more than 1.5 and with BH-adjusted p-value< = 0.05 were considered.

### Cell type-specific regulation of genes with ERβ-selective ligands

**Figure 6:**
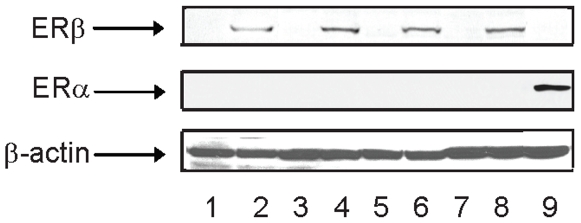
Immunoblot of ERα and ERβ in different cell lines. U2OS-ERβ cells in the absence (lane1) or presence of doxycycline (lane 2) was used as a positive control for ERβ. Ishikawa cells in the absence (lane 3) or presence (lane 4) of adenovirus (Ad)-ERβ, HeLa cells in the absence (lane5) or presence (lane 6) of Ad-ERβ, CaCo-2 cells in the absence (lane7) or presence (lane 8) of Ad-ERβ. U2OS-ERα cells in the presence of doxycycline (lane 9) was used as a positive control for ERα. ERα and ERβ were detected by immunoblotting with an ERα or ERβ antibodies.

**Figure 7:**
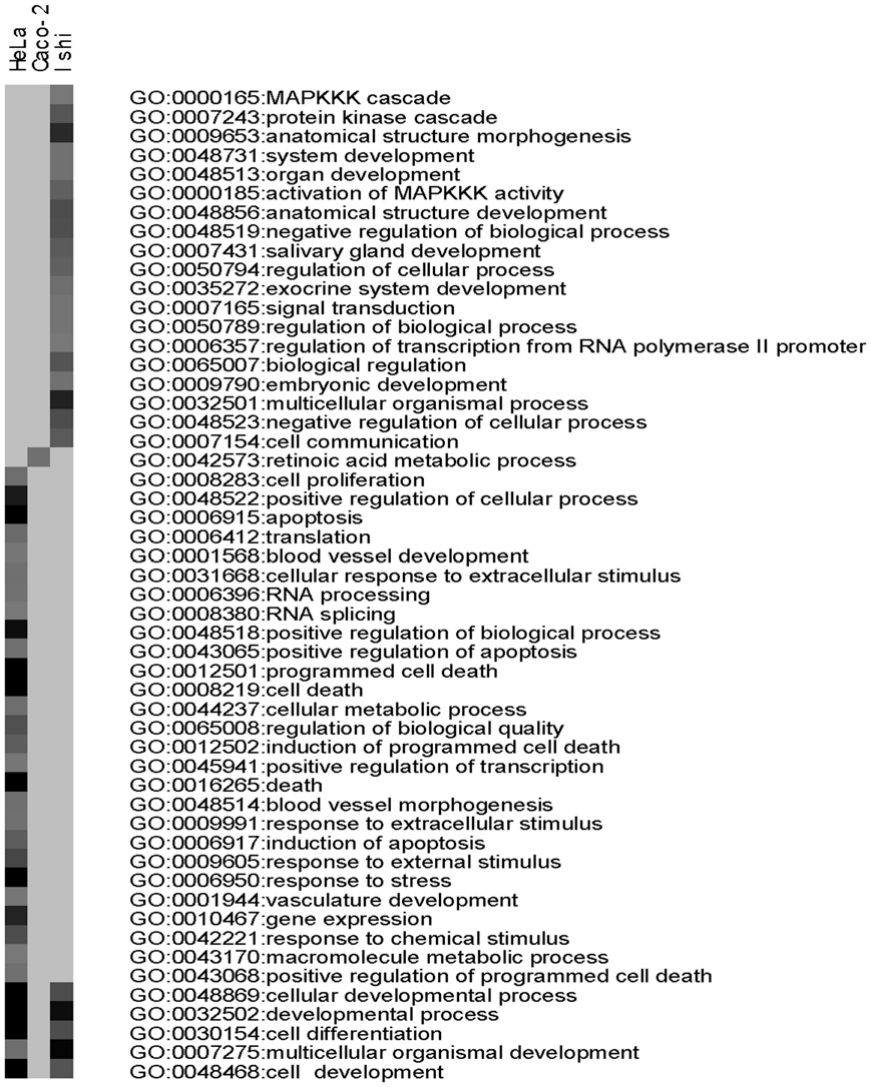
GO charts for genes regulated by MF101 in HeLa, Caco-2 or Ishikawa cells. Analysis of biological processes enriched among genes regulated by MF101 in HeLa, Caco-2 or Ishikawa (Ishi) cells. Gene ontology terms significantly enriched in genes regulated by MF101 in each of the four cell lines are shown. A threshold 0.001 was used for selecting GO terms using BH-adjusted p-values. 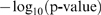 was used as an enrichment score. Darker shading denotes more significantly enriched GO terms, whereas the lightest gray implies the corresponding GO term is not significantly enriched.

**Figure 8:**
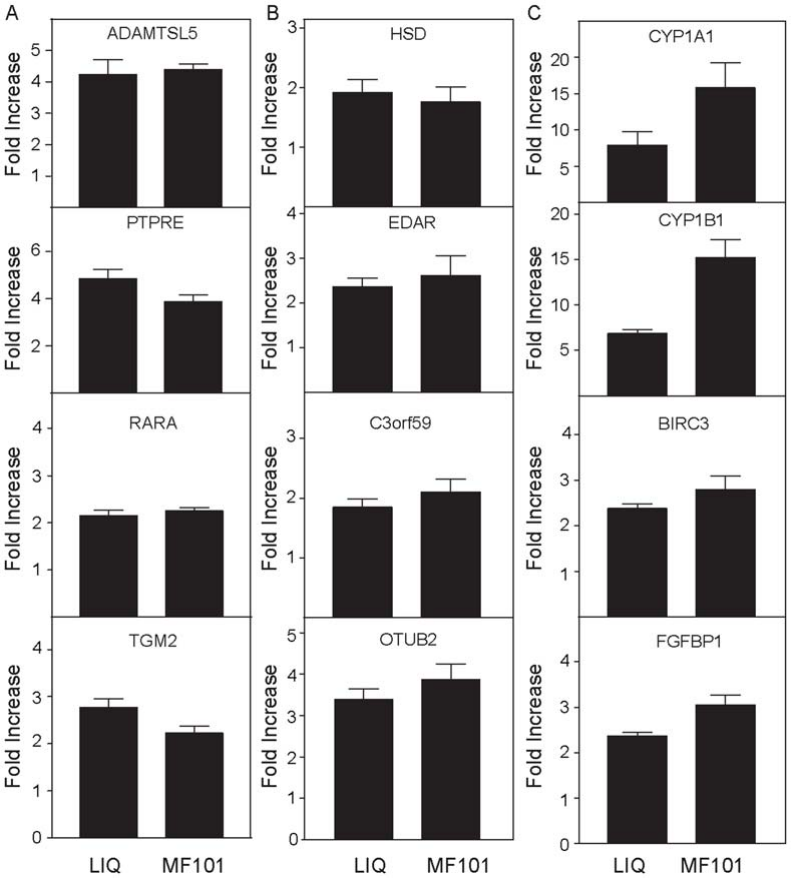
MF101 and LIQ regulation of selected genes in HeLa, Caco-2 or Ishikawa cells. HeLa (A), Ishikawa (Ishi, B) or Caco-2 (C) cells were infected with an adenovirus that expresses ERβ for 24 h. The cells were then treated with 1 µM LIQ or 125 µg/ml MF101 for 6 h. Real-time PCR was done to measure mRNA levels of ADAMTS-like 5 (ADAMTSL5), protein tyrosine phosphatase, receptor type, E (PTPRE), retinoic acid receptor, alpha (RARA), and transglutaminase 2 (TGM2) genes in HeLa cells (A), hydroxysteroid (11-beta) dehydrogenase 2 (HSD), ectodysplasin-A receptor (EDAR), chromosome 3 open reading frame 59 (C3orf59) and OTU domain, ubiquitin aldehyde binding 2 (OTUB2) in Ishikawa cells (B), cytochrome P450, family 1, subfamily A, polypeptide 1, (CYP1A1), cytochrome P450, family 1, subfamily B, polypeptide 1 (CYP1B1), baculoviral IAP repeat-containing 3 (BIRC3) and fibroblast growth factor binding protein 1 (FGFBP1) in Caco-2 cells (C). Each data point is the average of triplicate determinations. Error bars represent the mean±S.E.M.

To examine whether the ERβ-selective ligands regulate genes in a cell-specific manner, Caco-2, HeLa and Ishikawa cells were infected with an adenovirus that expresses ERβ. These three cell lines did not express ERα or ERβ (Figure 6). The expression of ERβ after the cells were infected with Ad-ERβ was similar in the three cell lines. For microarray analysis, we chose to focus on MF101 and LIQ, because this allowed us to evaluate if the effects of a crude extract were similar to a single active compound. The cells were treated for 6 h with MF101 or LIQ and the gene expression profiles were determined. Surprisingly, there was very little overlap in the regulated genes in the three cell lines (Table 3). Only 3 genes were commonly regulated by MF101 and no genes were commonly regulated by LIQ in the three cell types. Because only a few genes were commonly regulated by MF101 and LIQ in three cell lines, we compared the number of genes commonly regulated by these drugs in two cell lines (Table 4). The most overlap with MF101 treatment occurred in the Caco-2 and HeLa cells with 17 genes commonly regulated. The list of the regulated genes by MF101 or LIQ in three cell lines is found in Table S3. The GO analysis showed that not only do MF101 (Figure 7) and LIQ (Figure S5) regulate different genes, but also that the regulated genes are involved with different biological processes. These data demonstrate that there is a remarkable cell-type specificity of genes regulated by two of the ERβ-selective agonists. We used real-time PCR to examine the regulation by MF101 or LIQ in the three cell lines infected with Ad-ERβ. MF101 or LIQ increased mRNA levels for ADAMTS-like 5 (ADAMTSL5), protein tyrosine phosphatase, receptor type, E (PTPRE), retinoic acid receptor, alpha (RARA), and transglutaminase 2 (TGM2) genes in HeLa cells (Figure 8A), hydroxysteroid (11-beta) dehydrogenase 2 (HSD), ectodysplasin-A receptor (EDAR), chromosome 3 open reading frame 59 (C3orf59) and OTU domain, ubiquitin aldehyde binding 2 (OTUB2) in Ishikawa cells (Figure 8B), cytochrome P450, family 1, subfamily A, polypeptide 1, (CYP1A1), cytochrome P450, family 1, subfamily B, polypeptide 1 (CYP1B1), baculoviral IAP repeat-containing 3 (BIRC3) and fibroblast growth factor binding protein 1 (FGFBP1) in Caco-2 cells (Figure 8C). These results confirm the regulation observed in the microarrays.

**Table 3:** Summary of genes regulated by MF101 or LIQ in three cell lines.

	Number of Regulated genes	
	MF101	LIQ
Ishi	134	119
HeLa	382	70
Caco-2	88	31
Caco-2+HeLa	27	5
Ishi+Caco-2	0	1
Ishi+HeLa	6	4
Ishi+Caco-2+HeLa	3	0

Total genes uniquely regulated by the compounds in each cell line, or specifically regulated in different combinations of cell lines. Numbers are the probe set counts. HeLa, Caco-2, or Ishikawa cells were infected with an adenovirus that expresses ERβ for 24 h and were then treated with 125 µg/ml MF101 and 1 µM LIQ for 6 h. Microarrays were performed with WG-6 BeadChips. Genes with fold change more than 1.5 and with BH-adjusted p-value< = 0.05 were considered.

**Table 4:** Summary of regulated genes between cell pairs.

Drug	Cell 1	Cell 2	Specific to Cell 1	Specific to Cell 2	Common
MF101	Ishi	Caco-2	141	116	2
MF101	Ishi	Hela	138	413	5
MF101	Caco-2	Hela	101	401	17
LIQ	Ishi	Caco-2	123	36	1
LIQ	Ishi	Hela	122	77	2
LIQ	Caco-2	Hela	35	77	2

Numbers are the counts of probe sets regulated by MF101 or LIQ in HeLa, Caco-2 or Ishikawa (Ishi) cells. Genes with fold change more than 1.5 and with BH-adjusted p-value< = 0.05 were considered. Microarrays were performed with WG-6 BeadChips.

## Discussion

The biological effects of estrogens are mediated by ERα and ERβ. All the current estrogens approved for hormone therapy non-selectively bind to and regulate both ERs. ERα has an important role in preventing osteoporosis, because males with a defective ERα develop severe osteoporosis and the increased bone turnover is not reversed by high-dose estrogen treatment [42]. However, the activation of ERα by estrogens also causes the proliferation of cells, which increases the risk of breast and endometrial cancer [18]. The pro-proliferative properties of non-ER selective estrogens has prevented their use in non-hysterectomized women, and caused an intense effort to discover more selective estrogens. Drugs that selectively activate ERβ are a particularly attractive alternative for HT, because ERβ acts as a tumor suppressor that inhibits the growth of breast cancer cells [21], [22], [23]. The lack of proliferative effects of ERβ were also demonstrated by the observations ERB-041 did not exhibit any proliferative effects on the mammary glands and uterus of rats [40], and MF101 and LIQ did not stimulate uterine growth or breast cancer tumor formation in a mouse xenograft model [26], [27]. Whereas these results indicate that ERβ-selective agonists will not elicit the same proliferative effects as the non-selective estrogens, it is unclear if they will be beneficial for treating menopausal symptoms or osteoporosis.

Some ERβ-selective compounds did not show any benefits on hot flashes in rat models indicating that ERβ-selective agonists might not be effective for this classical indication for HT [43]. In contrast, DPN reduced hot flashes as measured by a reversal of the elevation in of basal tail skin temperature that occurs after ovariectomy [44]. The ERβ-selective agonist MF101 showed a statistically significant reduction in hot flashes in a phase 2 randomized placebo controlled study [45]. One possible explanation for these findings is that different classes of ERβ-selective agonists might regulate distinct genes and thereby produce different biological effects. To examine this possibility, we compared the ERβ-selectivity of synthetic and plant-derived ERβ-selective agonists in U20S cells that express ERα or ERβ using microarrays to study their selectivity over a broad range of ER target genes. We found that ERB-041, LIQ and MF101 were the most ERβ-selective, followed by NYA, and DPN.

The precise mechanism for the ERβ-selectivity of the compounds is unclear. ERB-041 is considered to be an ERβ-selective agonist because it binds to ERβ with about a 200-fold higher affinity compared to ERα [40]. DPN has a 70-fold higher affinity to ERβ, whereas LIQ bound to ERβ with a 20-fold higher affinity [27]. MF101 and NYA bound to ERα and ERβ with a similar affinity [26]. All of these binding studies used *in vitro* competition binding assays. To explore the relative binding of the compounds in living cells, we performed FRET studies in U2OS cells. Our FRET studies showing that ERB-041 was the only compound that did not produce any conformational change in ERα at 1 µM demonstrated that ERB-041 is a selective ERβ binder. In contrast, conformational changes in ERα and ERβ were induced at similar concentrations with MF101, LIQ, NYA and DPN, demonstrating that these compounds can bind to both ERα and ERβ. However, the gene expression data showed that even though they bound similar to ERα and ERβ at 1 µM, these compounds regulated genes selectively with ERβ at this concentration. These results indicate that the conformation of ERα induced by MF101 and LIQ is essentially inactive, whereas the conformation induced by NYA and DPN was weakly active. It is clear that at saturating levels the ERβ-selectivity of these compounds is not related to differential binding to ERβ, but results from events that occur after ligand binding. We previously showed that MF101 and LIQ did not recruit coactivators to ERα [26], [27], suggesting that compound-bound ERα was in a conformation that was incapable of binding coactivators. Our FRET data shows that the conformations produced by all ERβ-selective agonists were similar despite that they showed different patterns of gene regulation. The FRET study measures the position of YFP relative to CFP, which appeared to be very similar when ERβ is bound with the different compounds. It is likely that FRET is not sensitive enough to detect subtle changes in conformation that led to the differences in gene expression profiles with the compounds.

One of the most interesting findings of our study is that some genes regulated by the ERβ-selective compounds were not regulated by E_2_ in the U2OS-ERβ cells. The number of genes differentially regulated by the ERβ agonists compared to E_2_, range from 31 with DPN to 168 with MF101. These results demonstrate that the ERβ-selective compounds do not entirely mimic the action of E_2_ after binding to ERβ, suggesting that they might elicit different biological effects than E_2_. While there was no difference in FRET with E_2_ and the ERβ-selective compounds it is likely that subtle differences in conformation not detectable by FRET might lead to a differential recruitment of coregulatory proteins and ultimately different genes regulated. This issue is difficult to address experimentally because the regulatory elements in the genes that are differentially regulated by E_2_ and ERβ-selective agonists as observed with the microarrays are not known.

Our study also demonstrated that two ERβ-selective compounds regulated different genes in the three cell lines. Although the cells were exposed to the same amount of Ad-ERβ, concentration of drugs, and time of drug treatment there was very little overlap in the regulated genes in the these cell lines. Unexpectedly, only 3 genes were commonly regulated in all cell types. The reason for the cell-specific regulation is unknown. It has been proposed that the differential expression of coactivators in different cell types might be responsible for cell-specific regulation [46], [47]. Our microarrays showed similar expression of SRC-1, SRC-2 and SRC-3 in the three cell lines (data not shown). These findings indicate that the differential expression of these three classes of coactivators is an unlikely explanation for the different pattern of gene regulation in the cell lines. Genome-wide tiling arrays demonstrate that ER binding sites are associated with different transcription factors that are important for gene activation [48], [49], [50]. We also showed that the activation of the *NKG2E* gene requires multiple transcription factors (32). These findings suggest that differential expression of transcription factors in the cells might lead to the differences in gene regulation. Another explanation is that there are different epigenetic changes in the regulated genes in each cell type that allow the recruitment of cell specific transcription factors as shown with FOXA1 [51]. It is also possible that the drugs are differentially metabolized in the three cells. If the metabolites are active this might account for some of the differences in the genes regulated.

Our study shows several important features of ERβ-selective agonists that could have important clinical ramifications. First, although most of the genes regulated by the three different classes of ERβ-selective agonists were the same, there were some classes of genes that were differentially regulated and the magnitude of regulation of some regulated genes differed. These findings suggest that different ERβ-selective drugs might exert distinct clinical effects and that it can not be assumed that if one drug fails or succeeds in clinical trials that other ERβ-selective drugs will behave similarly. Second, the ERβ-selective agonists regulate different genes than E_2_. These findings suggest that ERβ-selective agonists will have a different side-effect profile than currently hormone therapy regimens. Although the effect of the ERβ-selective compounds on thromboembolic events is unknown, their benign effect on the uterus and mammary gland in preclinical models is a potentially differentiating factor from the non-selective estrogens. Our hypothesis that different classes of ERβ-selective agonists will produce distinct biological effects needs to be tested in clinical trials with postmenopausal women.

## Supporting Information

Figure S1 Structures of the compounds used.
(0.21 MB TIF)

Figure S2 Transfection Assays. U2OS cells were transfected with ERE-tKLuc and an expression vector for ERβ. The cells were treated for 18 h with increasing concentrations of NYA, DPN and ERB-041. Each data point is the average of triplicate determinations. Error bars represent the mean±S.E.M.
(0.21 MB TIF)

Figure S3 Analysis of biological processes enriched among ERβregulated genes between E2 and other compounds. Gene ontology (GO) terms showing significantly enriched in genes regulated by E2 or other compounds in U2OS-ERβ cells. A threshold 0.001 was used for selecting GO terms using BH-adjusted p-values. (p-value) was used as an enrichment score. Darker shading denotes more significantly enriched GO terms, whereas the lightest gray implies the corresponding GO term is not significantly enriched.
(0.44 MB TIF)

Figure S4 Analysis of biological processes commonly enriched among ERβ regulated genes between E2 and other compounds. Gene ontology (GO) terms showing significantly enriched in genes regulated by E2 or other compounds in U2OS-ERβ cells. A threshold 0.001 was used for selecting GO terms using BH-adjusted p-values. (p-value) was used as an enrichment score. Darker shading denotes more significantly enriched GO terms, whereas the lightest gray implies the corresponding GO term is not significantly enriched. GO terms significantly enriched in at least three conditions are shown.
(0.55 MB TIF)

Figure S5 GO charts for genes regulated by LIQ in HeLa, Caco-2 or Ishikawa cells. Analysis of biological processes enriched among genes regulated by LIQ in HeLa, Caco-2 or Ishi cells. Gene ontology terms significantly enriched in genes regulated by LIQ in each of the fours cell lines are shown. A threshold 0.001 was used for selecting GO terms using BH-adjusted p-values. (p-value) was used as an enrichment score. Darker shading denotes more significantly enriched GO terms, whereas the lightest gray implies the corresponding GO term is not significantly enriched.
(0.27 MB TIF)

Table S1 PCR Primer sequences used for real-time PCR.
(0.02 MB XLS)

Table S2 Genes regulated for each compound in U2OS-ERα, U2OS-ERβ cells or both U2OS-ERα and U2OS-ERβ cells.
(0.29 MB XLS)

Table S3 Genes regulated by MF101 and liquiritigenin in HeLa, Caco-2 or Ishikawa cells infected with an adenovirus that expresses ERβ.
(0.08 MB XLS)
