# Supplementary figures and images for: Drug and Cell Type-Specific Regulation of Genes with Different Classes of Estrogen Receptor β-Selective Agonists
Source: PLoS One. 2009 Jul 17;4(7):e6271. doi: 10.1371/journal.pone.0006271 (PMC2707612; doi:10.1371/journal.pone.0006271)

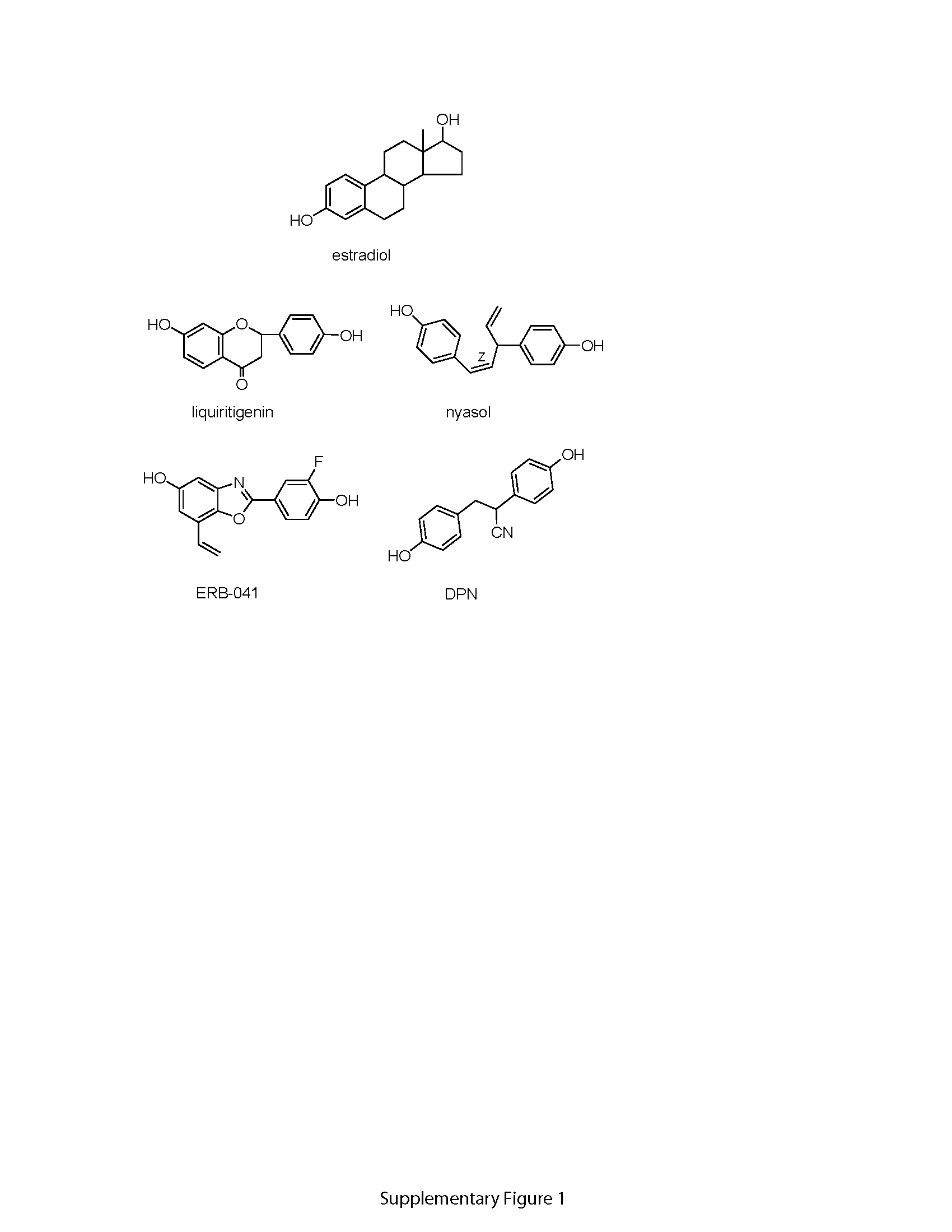

Supplement: Figure S1 — Structures of the compounds used. (0.21 MB TIF) [file pone.0006271.s001.tif]

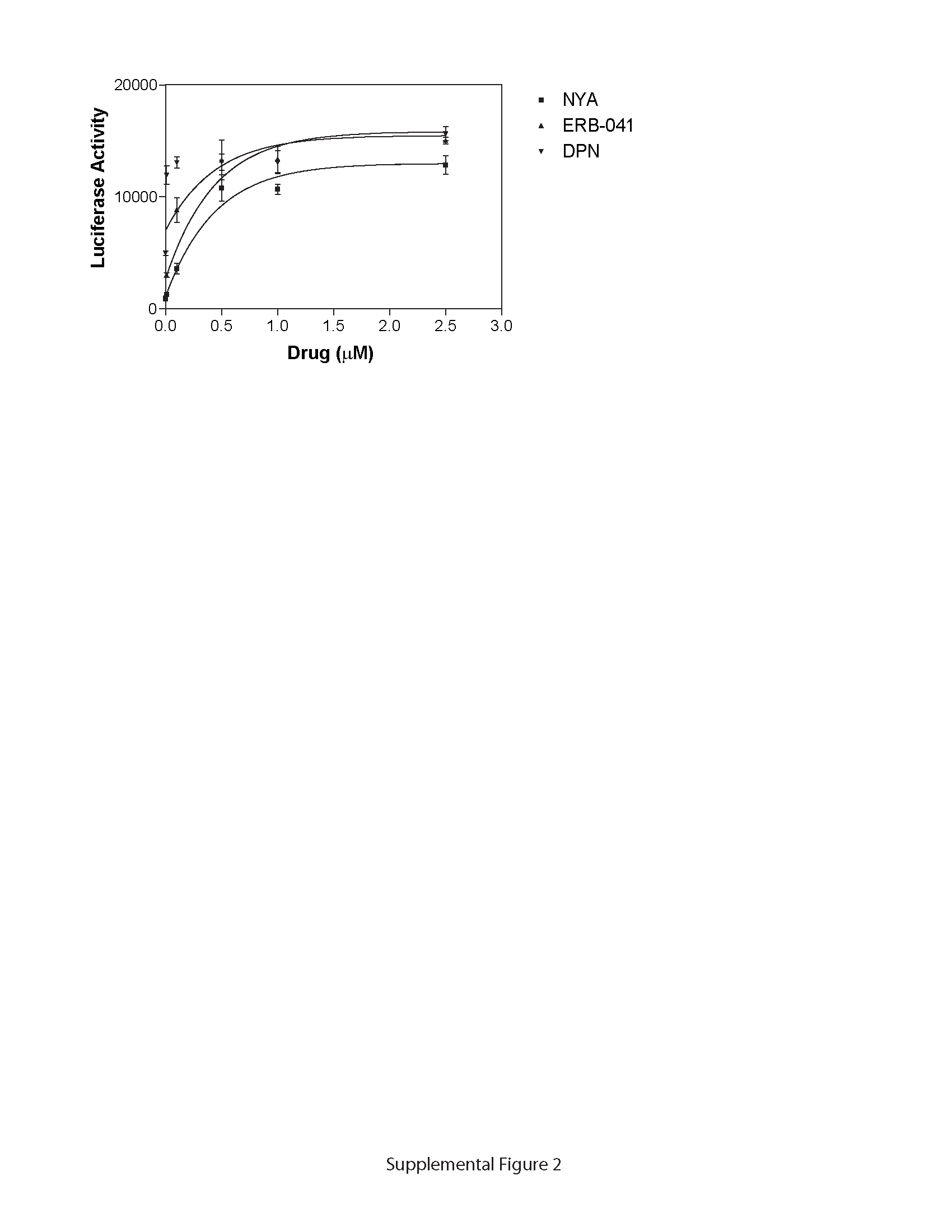

Supplement: Figure S2 — Transfection Assays. U2OS cells were transfected with ERE-tKLuc and an expression vector for ERβ. The cells were treated for 18 h with increasing concentrations of NYA, DPN and ERB-041. Each data point is the average of triplicate determinations. Error bars represent the mean±S.E.M. (0.21 MB TIF) [file pone.0006271.s002.tif]

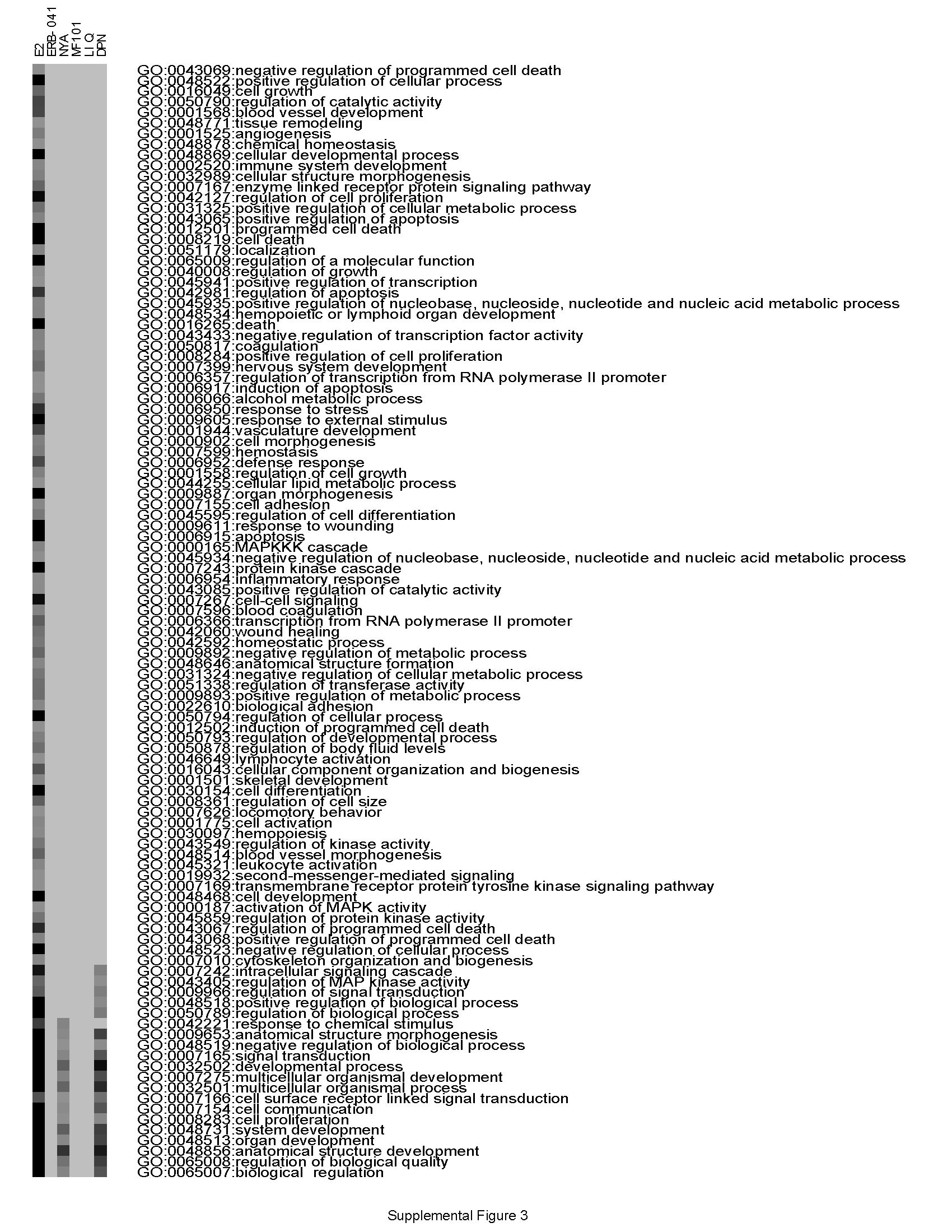

Supplement: Figure S3 — Analysis of biological processes enriched among ERβregulated genes between E2 and other compounds. Gene ontology (GO) terms showing significantly enriched in genes regulated by E2 or other compounds in U2OS-ERβ cells. A threshold 0.001 was used for selecting GO terms using BH-adjusted p-values. (p-value) was used as an enrichment score. Darker shading denotes more significantly enriched GO terms, whereas the lightest gray implies the corresponding GO term is not significantly enriched. (0.44 MB TIF) [file pone.0006271.s003.tif]

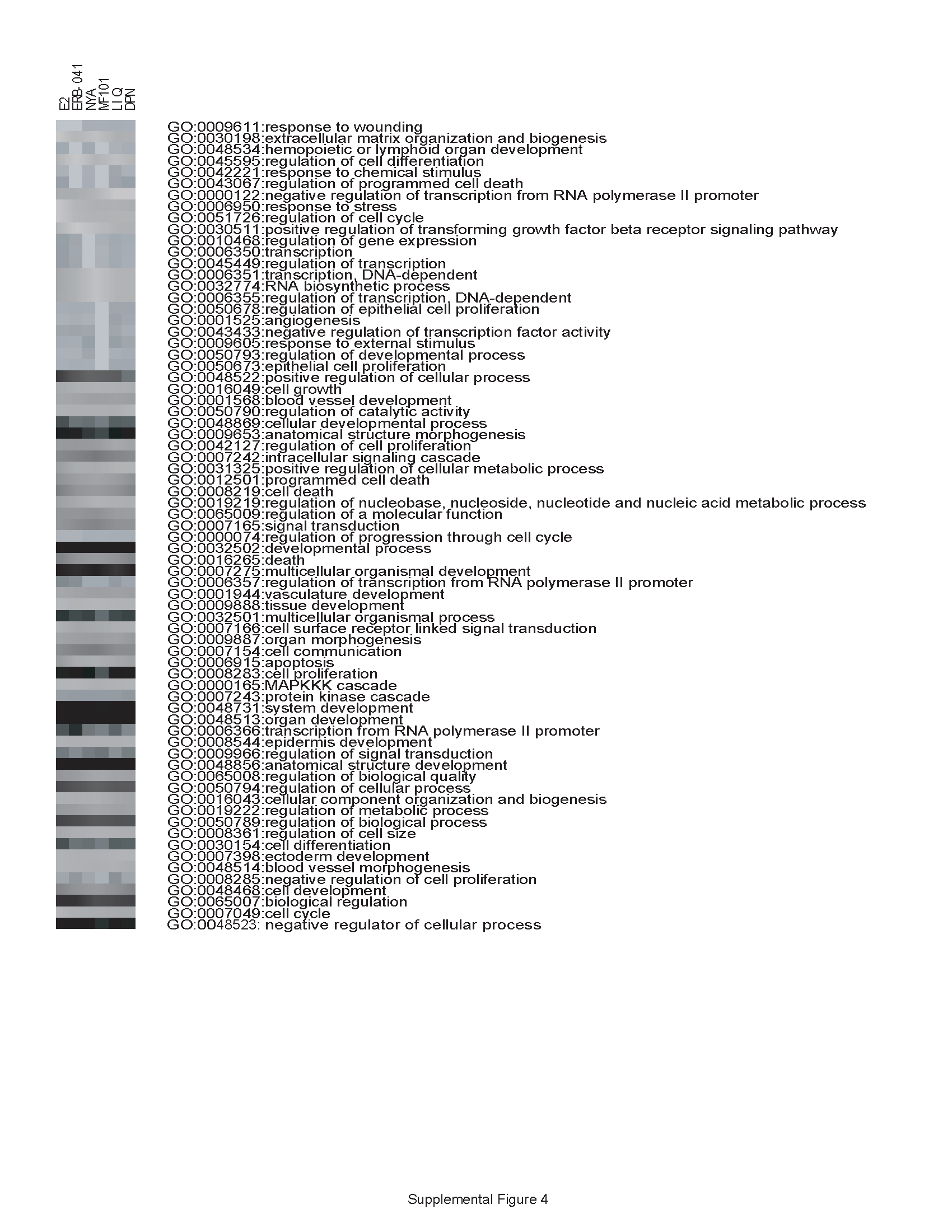

Supplement: Figure S4 — Analysis of biological processes commonly enriched among ERβ regulated genes between E2 and other compounds. Gene ontology (GO) terms showing significantly enriched in genes regulated by E2 or other compounds in U2OS-ERβ cells. A threshold 0.001 was used for selecting GO terms using BH-adjusted p-values. (p-value) was used as an enrichment score. Darker shading denotes more significantly enriched GO terms, whereas the lightest gray implies the corresponding GO term is not significantly enriched. GO terms significantly enriched in at least three conditions are shown. (0.55 MB TIF) [file pone.0006271.s004.tif]

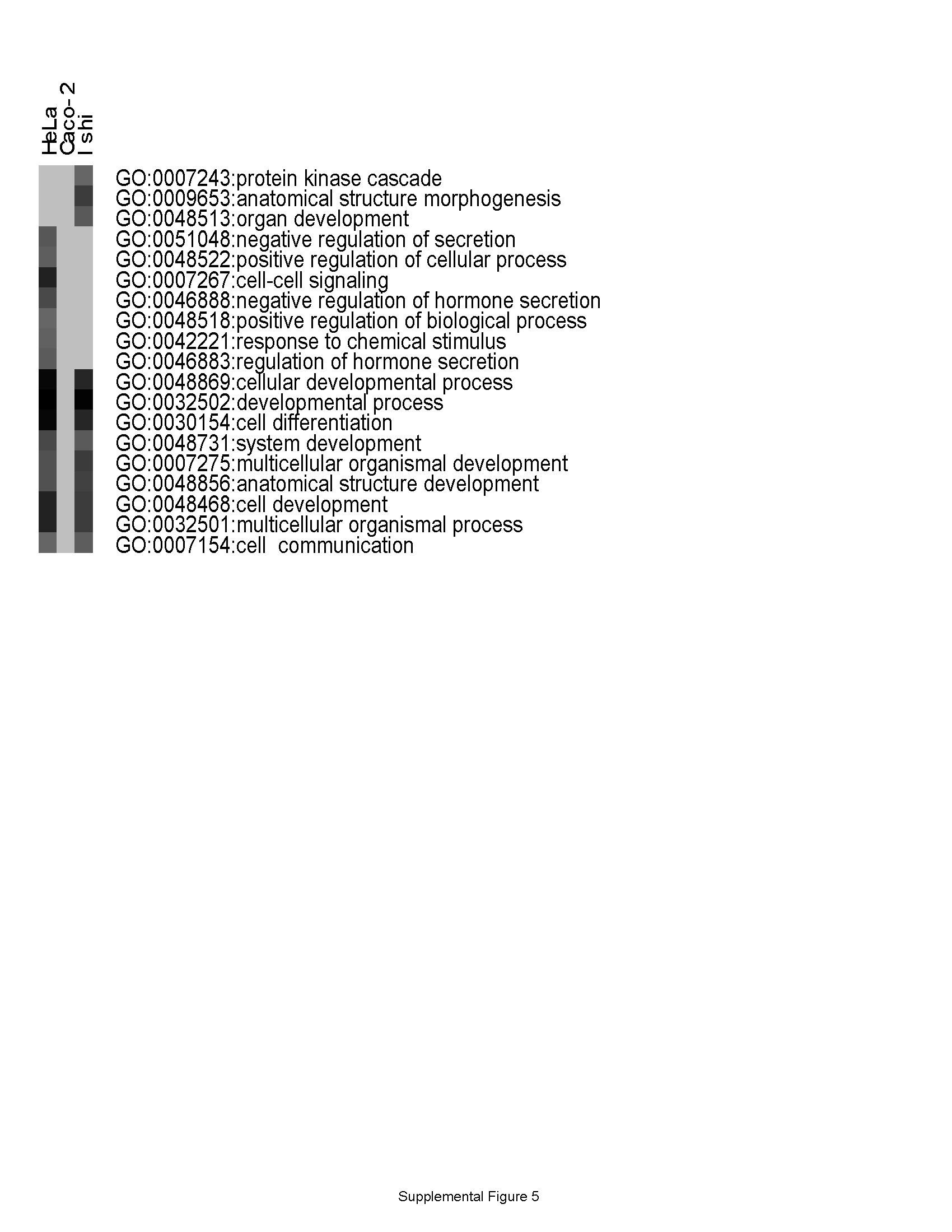

Supplement: Figure S5 — GO charts for genes regulated by LIQ in HeLa, Caco-2 or Ishikawa cells. Analysis of biological processes enriched among genes regulated by LIQ in HeLa, Caco-2 or Ishi cells. Gene ontology terms significantly enriched in genes regulated by LIQ in each of the fours cell lines are shown. A threshold 0.001 was used for selecting GO terms using BH-adjusted p-values. (p-value) was used as an enrichment score. Darker shading denotes more significantly enriched GO terms, whereas the lightest gray implies the corresponding GO term is not significantly enriched. (0.27 MB TIF) [file pone.0006271.s005.tif]
